# Methiocarb Degradation by Electro-Fenton: Ecotoxicological Evaluation

**DOI:** 10.3390/molecules25245893

**Published:** 2020-12-12

**Authors:** Faléstine Souiad, Ana Sofia Rodrigues, Ana Lopes, Lurdes Ciríaco, Maria José Pacheco, Yasmina Bendaoud-Boulahlib, Annabel Fernandes

**Affiliations:** 1 FibEnTech-UBI, Department of Chemistry, Universidade da Beira Interior, 6201-001 Covilhã, Portugal; souiad.falestine@ubi.pt (F.S.); asfrodrigues@hotmail.com (A.S.R.); analopes@ubi.pt (A.L.); lciriaco@ubi.pt (L.C.); mjap@ubi.pt (M.J.P.); 2 Département de Chimie, Faculté des Sciences Exactes, Université Constantine 1, 25000 Constantine, Algerie; boulahlib_yasmina@yahoo.fr

**Keywords:** emerging contaminants, electro-Fenton process, boron-doped diamond anode, carbon-felt, *Daphnia Magna*, acute toxicity

## Abstract

This paper studies the degradation of methiocarb, a highly hazardous pesticide found in waters and wastewaters, through an electro-Fenton process, using a boron-doped diamond anode and a carbon felt cathode; and evaluates its potential to reduce toxicity towards the model organism *Daphnia magna*. The influence of applied current density and type and concentration of added iron source, Fe_2_(SO_4_)_3_·5H_2_O or FeCl_3_·6H_2_O, is assessed in the degradation experiments of methiocarb aqueous solutions. The experimental results show that electro-Fenton can be successfully used to degrade methiocarb and to reduce its high toxicity towards *D. magna*. Total methiocarb removal is achieved at the applied electric charge of 90 C, and a 450× reduction in the acute toxicity towards *D. magna*, on average, from approximately 900 toxic units to 2 toxic units, is observed at the end of the experiments. No significant differences are found between the two iron sources studied. At the lowest applied anodic current density, 12.5 A m^−2^, an increase in iron concentration led to lower methiocarb removal rates, but the opposite is found at the highest applied current densities. The highest organic carbon removal is obtained at the lowest applied current density and added iron concentration.

## 1. Introduction

Water contamination by pesticides and herbicides is currently a major environmental concern, due to the toxic properties of these compounds and to their high potential to cause ecologically adverse effects [1,2,3]. Although the presence of these compounds in surface and ground waters is usually ascribed to their direct usage in the environment, the industrial production of pesticides and herbicides generates a large volume of wastewaters with a high concentration in these pollutants that, if not properly treated, will contaminate the freshwater bodies [4]. Many of the pesticides and herbicides are resistant to conventional treatment processes, and thus, the development of new treatment technologies, capable of degrading these contaminants into non-toxic and biodegradable products, is required [2,3]. Among these new treatment technologies, electrochemical processes have been widely studied, mainly due to their high efficiency in the degradation of recalcitrant compounds [3,5,6,7]. Silva et al. [8] achieved complete oxidation of the insecticide imidacloprid, at a concentration of approximately 25 mg L^−1^, by applying an anodic oxidation (AO) process with mixed metal oxide anodes. Successful degradation of the herbicides monolinuron, paraquat, and clopyralid and of the pesticide profenofos, at concentrations ranging 10–100 mg L^−1^, by electro-Fenton (EF), was also reported [2,9,10,11]. In fact, EF is a promising treatment process that promotes the electrooxidation of organic compounds through hydroxyl radicals produced in the Fenton reaction (Equation (1)), and, according to the literature, can present higher treatment efficiencies and mineralization indexes than AO [10,11].

Fe^2+^ + H_2_O_2_ → Fe^3+^ + ^•^OH + OH^−^ (1)

In the EF process, H_2_O_2_ can be continuously electrogenerated in situ (Equation (2)), and Fe^2+^ can be regenerated from the reduction of Fe^3+^ (Equation (3)), electrocatalyzing the reaction described by Equation (1), provided that a suitable cathode, like carbon-felt, is utilized [12,13]. Moreover, if a high O_2_-overpotential anode, such as boron-doped diamond (BDD), is applied, EF efficiency can be further enhanced by the additional organic matter oxidation through adsorbed hydroxyl radicals (BDD(^•^OH)), formed as an intermediate of the anodic water discharge [14,15].

O_2_ + 2H^+^ + 2e^−^ → H_2_O_2_ (2)

Fe^3+^ + e− → Fe^2+^ (3)

Although the degradation of different pesticides and herbicides by the EF process has been successfully described, few studies have addressed its ecotoxicological evaluation. The present study aims to contribute to this field, by evaluating the ecotoxicity of methiocarb (MC) solutions treated by EF.

MC is a carbamate pesticide, chemical formula C_11_H_15_NO_2_S, employed in agriculture worldwide, which has been detected in natural waters and in wastewaters [16,17,18,19]. MC is one of the priority substances identified by the Commission Implementing Decision (EU) 2018/840 watch list and classified as a highly hazardous pesticide by the World Health Organization, due to its high toxicity and some of its metabolites [20,21].

In a previous work [22], the ecotoxicity of MC aqueous solutions, with an initial MC concentration of 20 mg L^−1^, treated by AO, using a BDD anode, was evaluated towards the model organism *Daphnia magna*. A 200× reduction in the acute toxicity, from 370.9 to 1.6 toxic units, was observed after 5 h treatment at 100 A m^−2^, utilizing NaCl (250 mg L^−1^) as supporting electrolyte. It was found that, at the AO experimental conditions studied, NaCl-containing solutions led to faster MC degradation and higher toxicity reductions than Na_2_SO_4_-containing solutions. The explanation was that, in the chloride medium, the oxidation happened mainly in the bulk of the solution, and because the MC concentration was low, even at the lowest applied current density (100 A m^−2^) the active chlorine species formed were enough to oxidize MC at a very good reaction rate. Regarding the oxidation in the presence of sulfate, the mechanism occurred by hydroxyl and sulfate radicals, with smaller lifetime, thus giving more importance to MC diffusion towards the anode’s surface.

Since in the EF process, performed in the present work, hydroxyl radicals’ formation and subsequent organic matter oxidation can occur in the bulk of the solution, the hindrance caused by the pollutant molecules diffusion towards the anode’s surface is minimized, which can lead to higher treatment efficiencies than with AO.

MC degradation by electro-Fenton has never been reported. In fact, there are only a few studies addressing the application of electrochemical processes to degrade this compound [22,23]. Thus, the present work aimed to study the EF efficacy in the MC degradation and consequent reduction in toxicity towards *D. magna*. EF experiments of MC model aqueous solutions, at a concentration of 20 mg L^−1^, were carried out, and the influence of the type (iron sulfate or ferric chloride) and concentration (10 or 30 mg L^−1^) of iron source and of applied anodic current density (12.5, 25 or 50 A m^−2^), *j*, in the degradation of MC was evaluated, and a consequent reduction in toxicity towards *D. magna* was also assessed.

## 2. Results and Discussion

The characterization of the methiocarb aqueous solutions used in this study is presented in Table 1. In both MC solutions, containing iron sulfate or ferric chloride, the toxicity was very high, although the solution prepared with ferric chloride was slightly less toxic. The initial pH of the prepared solutions was around 3, which is the optimum pH for the EF process, due to its influence on iron speciation and on H_2_O_2_ decomposition [12].

**Figure 1:**
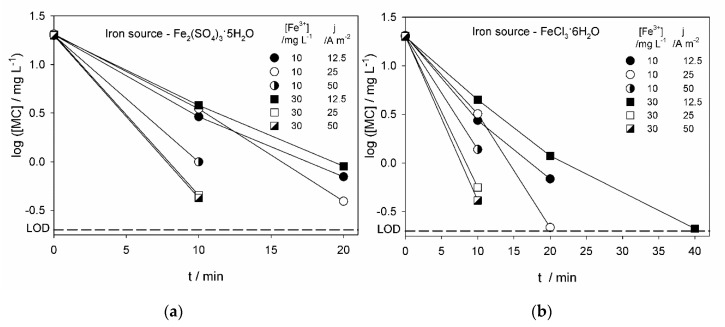

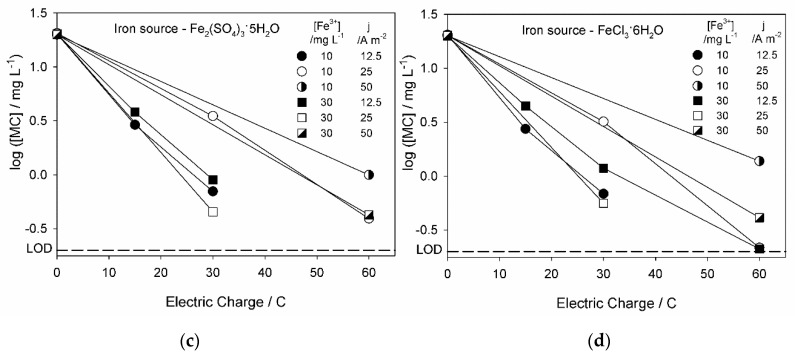
Methiocarb concentration logarithm decay with (**a**,**b**) time and with (**c**,**d**) applied charge for the electro-Fenton (EF) experiments performed with (**a**,**c**) Fe_2_(SO_4_)_3_·5H_2_O and (**b**,**d**) FeCl_3_·6H_2_O, at different iron concentrations and applied current densities. LOD, limit of detection.

Figure 1 presents the logarithm of the MC concentration decay with time (Figure 1a,b) for the EF experiments performed with different iron sources and concentrations and applied anodic current densities. A logarithmic scale was chosen because MC concentration decay was very abrupt during the first minutes of the assay. MC concentration decay was also analyzed as a function of the applied electric charge (Figure 1c,d), so conclusions could be drawn regarding the energetic efficiency of the EF process at the different experimental conditions.

Results presented in Figure 1 were obtained from EF experiments with 1 h duration, being samples collected every 10 minutes for methiocarb concentration determination. Only the results above the methiocarb limit of detection (LOD = 0.198 mg L^−1^) are presented in Figure 1, meaning that for the samples collected at longer times, MC was not detected.

For all the experimental conditions studied, no MC was detected in solution after 40 minutes of assay or applied electric charge of 120 C, meaning that at least 99% of the initial MC was degraded. Also, no significant differences in MC removals were found when using iron sulfate or ferric chloride.

At the iron concentration of 10 mg L^−1^, electric charge efficiency regarding MC removal decreased with the increase in *j*. In fact, according to the literature, the increase in applied current density would lead to an increase of secondary reactions, such as the described by Equations (4) and (5), promoted by the increased H_2_O_2_ production (Equation (2)) and hydroxyl radicals at the anode surface by water discharge [25]. Consequently, MC degradation efficiency would decrease.

OH + H_2_O_2_ → HO^•^_2_ + H_2_O (4)

HO^•^_2_ + ^•^OH → H_2_O + O_2_ (5)

When the iron concentration was increased to 30 mg L^−1^, MC removal rate and electric charge efficiency decreased, at the lowest *j*, 12.5 A m^−2^, but increased at the highest currents, 25 and 50 A m^−2^. These results might be explained considering that, at the highest iron concentration and applied current densities, the reactions described by Equations (1)–(3) are enhanced, increasing the MC degradation through the hydroxyl radicals formed in Fenton´s reaction. Also, with the increase in iron salts concentration and applied current density, an increase in the electric potential difference between anode and cathode was observed, despite the small increase in the conductivity of the solution promoted by the iron salt addition (Supplementary Material, Table S1). Thus, due to this increase in potential, sulfate and chloride oxidation occurred in higher extension, increasing the formation of chloride and sulfate-based oxidant species and subsequent MC additional indirect oxidation by these species [26].

**Figure 2:**
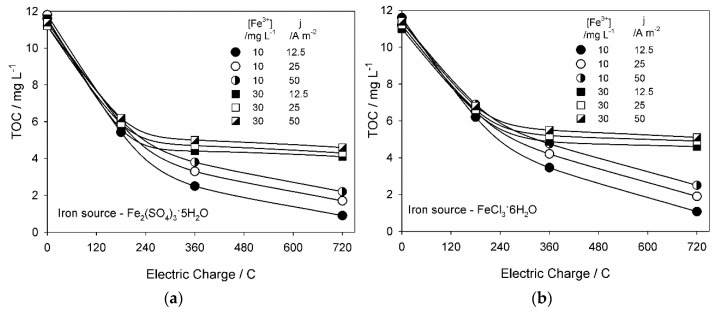
Total organic carbon (TOC) decay with applied charge for the EF experiments performed with (**a**) Fe_2_(SO_4_)_3_·5H_2_O and (**b**) FeCl_3_·6H_2_O, at different iron concentrations and applied current densities.

When total organic carbon (TOC) decay results are analyzed (Figure 2), it can be observed that, even for the highest removals achieved, after 720 C, there was still a residual TOC concentration in the treated solutions, indicating that the complete mineralization of MC degradation products was not accomplished. Still, TOC removals above 90%, corresponding to a final TOC concentration of approximately 1 mg L^−1^, were attained for the EF experiments performed using the lowest iron concentration (10 mg L^−1^) and applied current density (12.5 A m^−2^). For both iron sources and concentrations, TOC removal efficiency decreased with the increase in *j*, as observed for MC removal at 10 mg L^−1^ in iron, probably due to increased secondary reactions, as described above. Regarding the influence of iron concentration, for both iron sources, higher TOC removals were observed for the experiments performed with 10 mg L^−1^ of iron. In fact, for the solutions with 30 mg L^−1^ of iron, TOC removals attained similar efficiencies to those at 10 mg L^−1^ of iron until the applied electric charge of 200 C, but, after that, a plateau in TOC decay was observed, with no significant decrease in TOC concentration.

This lower TOC removal at higher added iron concentration is in agreement with the increased oxidation by chloride and sulfate-based oxidant species at these conditions, since, according to the literature, these species favor the organic compounds partial oxidation (conversion) rather than their complete mineralization, favored in the oxidation through ^•^OH [5]. The low or almost inexistent TOC removal observed in the last hours of assays might be a result of the formation of short-chain organic acids that are difficult to be further oxidized.

**Figure 3:**
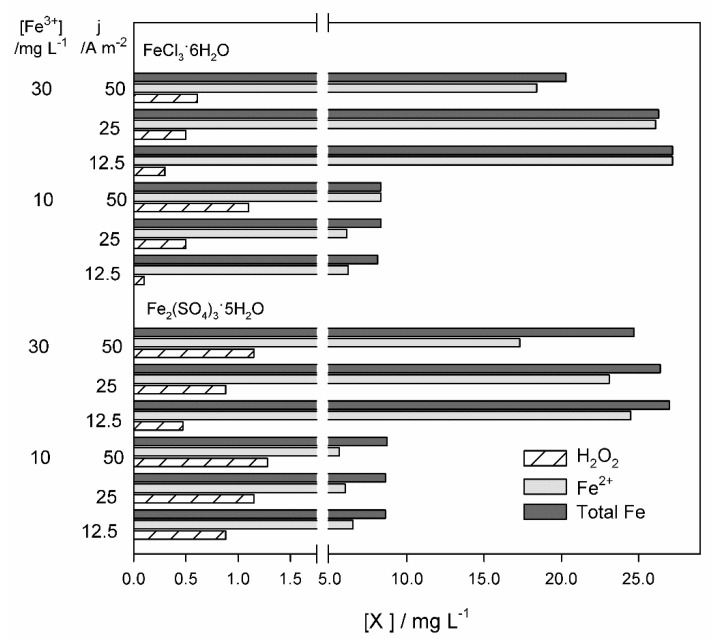
Hydrogen peroxide, dissolved Fe^2+^, and total dissolved iron concentrations at the end of the EF assays (applied electric charge = 720 C) performed with Fe_2_(SO_4_)_3_·5H_2_O and FeCl_3_·6H_2_O, at different applied current densities.

Although no great differences were observed between TOC removals using iron sulfate or ferric chloride, slightly higher removals were achieved when iron sulfate was utilized. However, when H_2_O_2_, total dissolved iron, and dissolved Fe^2+^ final concentrations are analyzed (Figure 3), it can be seen that the EF process was more effective in experiments performed with ferric chloride, since more of the total available iron was in the form of Fe^2+^ and lower H_2_O_2_ concentrations were found in solution, indicating its higher consumption. These results can indicate that, in iron sulfate-containing solutions, MC oxidation through hydroxyl radicals at the anode surface, produced from water discharge, occurs in higher extension than in solutions containing ferric chloride, resulting in higher TOC removals. From Figure 3, it can also be seen that the total dissolved iron concentration at the end of the experiments is always lower than the initial one and that, for the experiments performed with 30 mg L^−1^ of added iron, total dissolved iron concentration decreased with the increase of applied current density. According to the literature, this decrease in total dissolved iron concentration can be due to the formation of iron complexes [25].

**Figure 4:**
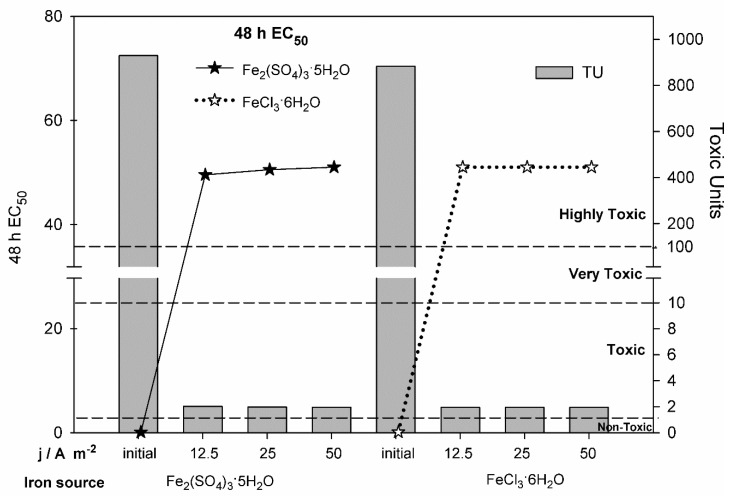
Toxicity towards *Daphnia magna* of the MC solutions prepared with 10 mg L^−1^ of iron, before and after the EF treatment at the different *j* studied, at the applied electric charge of 720 C, in terms of EC_50_ (represented by dots) and toxic units (represented by bars). The indication of the toxicity classification according to Pablos et al. [24] is also depicted (TU < 1 non-toxic; 1 < TU < 10: toxic; 10 < TU < 100: very toxic; TU > 100: highly toxic).

Since MC is a highly toxic compound and the presence of iron salts can also contribute to solution´s toxicity, the ecotoxicity of the initial and treated solutions was evaluated towards *D. magna*, a freshwater crustacean whose utilization as a model species in ecotoxicological tests is recommended by American Society for Testing and Materials (ASTM), Organization for Economic Cooperation and Development (OECD), and International Organization for Standardization (ISO). In fact, methiocarb (an acetylcholinesterase inhibitor pesticide, widely used in agriculture as a molluscicide, acaricide, and avicide) has already been studied in regards of its toxicity towards aquatic invertebrates [27]. Attending that, at the end of EF treatment, no methiocarb was detected in any of the experiments performed, and the higher TOC removals were achieved by the solutions containing 10 mg L^−1^ of iron—only solutions prepared with this iron amount were ecotoxicologically evaluated. Figure 4 presents the ecotoxicity results, in terms of EC_50_ and toxic units, after 48 h of exposure, of the MC solutions prepared with 10 mg L^−1^ of iron, before and after the EF treatment at the different *j* studied, at the applied electric charge of 720 C. Results show that EF treatment of methiocarb solutions led to a remarkable decrease in the acute toxicity towards *D. magna*. No meaningful differences were found in the ecotoxicity results of the treated samples evaluated. For iron sulfate-containing solutions, the increase in EC_50_ was from 0.108% to 50.4%, on average, corresponding to an average toxicity reduction of 468×, from 929.4 TU to 1.99 TU. For the experiments performed with added ferric chloride, an increase in EC_50_ from 0.113% to 51.01% was achieved, corresponding to a toxicity reduction of 450×, from 883.1 TU to 1.96 TU. These results demonstrate the high EF treatment efficiency for MC toxicity removal. The MC solutions with added iron sulfate or ferric chloride, classified as highly toxic before the EF assay, became only toxic after the treatment and was almost considered non-toxic (TU < 1), according to the toxicity classification based on toxic units reported by Pablos et al. [24].

When comparing these results with the previously obtained by AO treatment [22], it can be seen that, despite the toxicity of the initial samples is significantly increased by the addition of iron salts, the EF process is able to degrade methiocarb and its by-products and to reduce the acute toxicity towards *D. magna* to values similar to those obtained with AO treatment, being a feasible alternative for the remediation of contaminated waters and wastewaters by pesticides.

## 3. Materials and Methods 

The methiocarb aqueous solutions were prepared with ultrapure water, obtained with Milli-Q system (Merck, Lisbon, Portugal), using methiocarb PESTANAL^®^, analytical standard (CAS Number 2032-65-7), purchased from Sigma-Aldrich (Lisbon, Portugal). Iron(III) sulfate pentahydrate, 97% (CAS Number 10028-22-5) and ferric chloride hexahydrate, 97% (CAS Number 10025-77-1), were also purchased from Sigma-Aldrich (Lisbon, Portugal). The composition of the different solutions studied is presented in Table 2.

The electro-Fenton experiments were conducted in batch mode, using an undivided and cylindrical glass cell, with a useful volume of 200 mL. During the experiments, the solution was continuously stirred, with a magnetic bar, to enhance the mass transport of reactants/products toward/from the electrodes. A carbon-felt piece (Carbone Loraine, Coventry, UK) with a thickness of 0.5 cm and an immersed area of 130 cm^2^ was used as cathode, and a BDD electrode, purchased from Neocoat (La Chaux-de-Fonds, Switzerland), with an immersed area of 20 cm^2^, was used as an anode. The anode was centered in the cell and surrounded by the cathode, which covered the inner wall of the cell. Continuous O_2_ saturation at atmospheric pressure was ensured by bubbling compressed air through a fritted glass diffuser at 1 L min^−1^, starting 10 min before the assay, to reach a steady O_2_ concentration that allowed H_2_O_2_ electrogeneration (Equation (2)). To ensure that H_2_O_2_ was being properly produced and was enough to react with iron, according to Equation (1), H_2_O_2_ determinations were performed.

The experimental setup utilized a DC power supply GW, Lab DC, model GPS-3030D (0–30 V, 0–3 A), purchased from ILC (Lisbon, Portugal), being the anodic current densities studied 12.5, 25, and 50 A m^−2^. At the anodic current density of 12.5 A m^−2^, experiments were performed with 2, 4, and 8 h duration (180, 360, and 720 C, respectively). To apply the same electric charge (180, 360, and 720 C) to the experiments run at 25 A m^−2^, these assays had 1, 2, and 4 h duration, respectively, and at 50 A m^−2^ had 0.5, 1, and 2 h duration. The initial and final solutions from each experiment were utilized for methiocarb, total carbon, inorganic carbon, total organic carbon, the total dissolved iron, dissolved Fe^2+^, H_2_O_2_, pH, and conductivity determinations, that were performed in triplicate. The toxicity towards *D. magna* was only assessed for the solutions containing 10 mg L^−1^ of iron, before and after the EF treatment at 720 C, for the different tested *j*.

Since after 2 h of assay no methiocarb was detected in any of the experiments performed, assays with 1 h duration were performed, for all the experimental conditions studied, with samples being collected every 10 minutes, for methiocarb concentration determination. The samples were collected from the reaction vessel utilizing a pipette, without interrupting the assay, and the volume collected in each sample (2 mL) was the minimum required to perform the analysis in triplicate.

All the described EF experiments were performed in duplicate, with reproducibility found in all the experimental conditions studied. Data presented in Table 1 and Figure 1, Figure 2 and Figure 3 correspond to the mean values obtained from the analysis results, in triplicate, of the two assays performed.

Methiocarb decay was followed by high-performance liquid chromatography (HPLC), using a Shimadzu 20A Prominence HPLC system, purchased from Izasa Scientific (Carnaxide, Portugal), equipped with an SPD-M20A diode array detector, a CTO-20AC column oven, and an LC-20AD pump. A Purospher STAR RP18 end-capped column (250 × 4 mm (i.d.), 5 μm), purchased from VWR International (Amadora, Portugal), was utilized, being the elution isocratically performed with a mixture of formic acid aqueous solution (0.05%) and acetonitrile, 50:50 (*v*/*v*), at a flow rate of 1 mL min^−1^ and 35 °C. The injection volume was 20 μL, and the detection wavelength was 262 nm. HPLC solutions were prepared using HPLC grade reagents, supplied by Sigma-Aldrich (Lisbon, Portugal), and ultrapure water, obtained with Milli-Q system (Merck, Lisbon, Portugal). Total carbon, inorganic carbon, and total organic carbon were measured in a Shimadzu TOC-V CPH analyzer, purchased from Izasa Scientific (Carnaxide, Portugal). Total dissolved iron and dissolved Fe^2+^ concentrations were determined using a spectrometric method with 1,10-phenanthroline, according to ISO 6332:1988 [28]. H_2_O_2_ concentration was determined by the colorimetric metavanadate method [29]. Ecotoxicity was evaluated, using a Daphtoxkit F microbiotest, DM190320 (MicroBioTests, Inc., Gent, Belgium), by measuring the number of immobilized *D. magna* neonates exposed to different dilutions of the different MC solutions, collected before and after EF experiments. The concentration responsible for 50% of immobilization, EC_50_, was calculated using the standard data processing method Daphtoxkit F spreadsheet (MicroBioTests, Inc., Gent, Belgium). The acute toxicity results were also expressed in terms of toxicity units, TU, according to Equation (6) [24].

TU = 100/(% EC_50_) (6)

pH was measured with a HANNA pH meter (HI 931400) and conductivity using a Mettler Toledo conductivity meter (SevenEasy S30K), both purchased from MT Brandão (Oporto, Portugal).

## 4. Conclusions

The electro-Fenton treatment, using a BDD anode and a carbon-felt cathode, can be successfully applied to degrade methiocarb and its by-products and reduces drastically the acute toxicity towards *D. magna*. In the presence of iron(III) sulfate pentahydrate, 10 mg L^−1^ in iron, at an applied current density of 12.5 A m^−2^, total MC removal (20 mg L^−1^) was accomplished at the applied electric charge of 60 C, and at the end of the treatment (720 C), TOC concentration and toxicity were reduced from 11.6 mg L^−1^ and 929.4 TU to, respectively, 0.9 mg L^−1^ and 2.02 TU. Although no substantial differences were found between Fe_2_(SO_4_)_3_ and FeCl_3_ as iron sources, higher TOC removals were achieved with iron sulfate, probably due to higher extension of organic’s oxidation through hydroxyl radicals at the anode surface, produced from water’s discharge. At the highest applied current densities (25 and 50 A m^−2^), MC removal rate increased with initial iron concentration, since at these conditions, Fenton’s reaction is enhanced and sulfate and chloride oxidation occurred in higher extension, increasing the formation of chloride and sulfate-based oxidant species. Despite this, the highest TOC removals were observed for the experiments performed with the lowest added iron concentration (10 mg L^−1^) and applied current density (12.5 A m^−2^), where the organic’s oxidation through hydroxyl radicals at the anode’s surface occurs with higher extension.

## Figures and Tables

**Table 1:** Characterization of the methiocarb aqueous solutions used in the experiments.

Parameter		MC + Iron Sulfate		MC + Ferric Chloride	
Fe^3+^/mg L^−1^		9.7 ± 0.7	30 ± 1	9.5 ± 0.9	29.6 ± 0.9
Methiocarb/mg L^−1^		20.3 ± 0.5	19.8 ± 0.3	19.8 ± 0.5	20.0 ± 0.6
Total carbon/mg L^−1^		11.8 ± 0.3	11.6 ± 0.2	11.7 ± 0.2	11.5 ± 0.2
Inorganic carbon/mg L^−1^		0.26 ± 0.02	0.21 ± 0.01	0.25 ± 0.01	0.28 ± 0.03
Total organic carbon/mg L^−1^		11.5 ± 0.3	11.4 ± 0.2	11.4 ± 0.2	11.2 ± 0.2
pH		3.34 ± 0.03	2.88 ± 0.01	3.27 ± 0.01	2.81 ± 0.02
Conductivity/µS cm^−1^		176 ± 9	504 ± 8	207 ± 1	549 ± 9
Acute toxicity/	EC_50_ (48 h)	0.108%	n.d. ^2^	0.113%	n.d. ^2^
	TU ^1^	929.4	n.d. ^2^	883.1	n.d. ^2^

^1^ TU-toxic units (TU = (1/EC_50_) × 100, TU > 100: highly toxic, 10 < TU < 100: very toxic, 1 < TU < 10: toxic, TU < 1 non-toxic [24]). ^2^ n.d., not determined.

**Table 2:** Composition of the methiocarb aqueous solutions used in the experiments.

Reagent	MC + Iron Sulfate		MC + Ferric Chloride	
	10 mg L^−1^ Fe	30 mg L^−1^ Fe	10 mg L^−1^ Fe	30 mg L^−1^ Fe
Methiocarb/mg L^−1^	20.2 ± 0.3	20.1 ± 0.2	20.2 ± 0.2	20.0 ± 0.1
Fe_2_(SO_4_)_3_·5H_2_O/mg L^−1^	45.1 ± 0.4	136.4 ± 0.3	-	-
FeCl_3_·6H_2_O/mg L^−1^	-	-	50.0 ± 0.3	149.8 ± 0.2

## Supplementary Materials

The following are available online, Table S1: Medium electric potential difference (U) values observed in the MC degradation assays.
