# Supplementary material for: Methiocarb Degradation by Electro-Fenton: Ecotoxicological Evaluation
Source: Molecules. 2020 Dec 12;25(24):5893. doi: 10.3390/molecules25245893 (PMC7763907; doi:10.3390/molecules25245893)
Supplement: Supplementary file 1 [file molecules-25-05893-s001.pdf]

Table S1. Medium electric potential difference (U) values observed in the MC degradation assays.

Electrolyte	[Fe] / mg L^{-1}	I / A	U / V
Iron sulfate	10	0.025	5.9
		0.05	11.4
		0.1	16.3
	30	0.025	4.8
		0.05	6.5
		0.1	10.3
Ferric chloride	10	0.025	16.2
		0.05	9.3
		0.1	6.6
	30	0.025	5.3
		0.05	8.4
		0.1	12.3
